# Diversity and Functional Evolution of Terpene Synthases in Rosaceae

**DOI:** 10.3390/plants11060736

**Published:** 2022-03-10

**Authors:** Aidi Zhang, Yuhong Xiong, Jing Fang, Xiaohan Jiang, Tengfei Wang, Kangchen Liu, Huixiang Peng, Xiujun Zhang

**Affiliations:** 1 Key Laboratory of Plant Germplasm Enhancement and Specialty Agriculture, Wuhan Botanical Garden, Chinese Academy of Sciences, Wuhan 430000, China; zhangaidi@wbgcas.cn (A.Z.); xiongyuhong20@mails.ucas.ac.cn (Y.X.); fangjing17@mails.ucas.ac.cn (J.F.); jiangxiaohan16@mails.ucas.ac.cn (X.J.); wangtengfei17@mails.ucas.ac.cn (T.W.); liukangchen18@mails.ucas.ac.cn (K.L.); penghuixiang20@mails.ucas.ac.cn (H.P.); 2 Center of Economic Botany, Core Botanical Gardens, Chinese Academy of Sciences, Wuhan 430074, China; 3 University of Chinese Academy of Sciences, Beijing 100049, China

**Keywords:** terpene synthases, Rosaceae, expansion, evolution

## Abstract

Terpenes are organic compounds and play important roles in plant development and stress response. Terpene synthases (TPSs) are the key enzymes for the biosynthesis of terpenes. For Rosaceae species, terpene composition represents a critical quality attribute, but limited information is available regarding the evolution and expansion occurring in the terpene synthases gene family. Here, we selected eight Rosaceae species with sequenced and annotated genomes for the identification of TPSs, including three *Prunoideae*, three *Maloideae*, and two *Rosoideae* species. Our data showed that the TPS gene family in the Rosaceae species displayed a diversity of family numbers and functions among different subfamilies. Lineage and species-specific expansion of the TPSs accompanied by frequent domain loss was widely observed within different TPS clades, which might have contributed to speciation or environmental adaptation in Rosaceae. In contrast to *Maloideae* and *Rosoideae* species, *Prunoideae* species owned less TPSs, with the evolution of *Prunoideae* species, TPSs were expanded in modern peach. Both tandem and segmental duplication significantly contributed to TPSs expansion. Ka/Ks calculations revealed that *TPSs* genes mainly evolved under purifying selection except for several pairs, where the divergent time indicated TPS-e clade was diverged relatively anciently. Gene function classification of TPSs further demonstrated the function diversity among clades and species. Moreover, based on already published RNA-Seq data from NCBI, the expression of most TPSs in *Malus domestica*, *Prunus persica*, and *Fragaria vesca* displayed tissue specificity and distinct expression patterns either in tissues or expression abundance between species and TPS clades. Certain putative TPS-like proteins lacking both domains were detected to be highly expressed, indicating the underlying functional or regulatory potentials. The result provided insight into the TPS family evolution and genetic information that would help to improve Rosaceae species quality.

## 1. Introduction

The Rosaceae family consists of more than 2500 species in more than 90 genera and the family is divided into four subfamilies based on fruits: *Spiraeoideae* (*Spirea* subfamily), *Rosoideae* (rose subfamily), *Prunoideae* (plum subfamily), and *Maloideae* (apple subfamily). A number of species in the Rosaceae family are of economic importance as food crops, such as peaches, apples, almonds, cherries, pears, raspberries, and strawberries. Some species in the Rosaceae family are grown as ornamentals, such as the spiraea and rose. For Rosaceae, the inner quality of fruits or flowers is mainly determined by aroma and flavor. The aroma components are mainly composed of volatile products and terpenoids are important components of the volatile products such as linalool, (E)-β–damarone, and β–ionone [1]. Terpenoid compounds have been characterized in almond, apple, and peach [2,3,4,5]. Volatile compounds in almond (plum subfamily) mainly consist of fatty acid-derived volatiles, several monoterpenes, sesquiterpenes, and phenylpropanoids, and significant differences in volatile composition were observed between different tissues and various varieties [3]. Apple (apple subfamily) fruit produce more than 300 volatile organic compounds (VOCs), including alcohols, aldehyde esters, and ketones; the specific VOC composition in apple depends on several factors, including cultivar, climacteric ethylene production levels, maturity, and environmental conditions [4,5]. More than 100 volatile chemicals have been identified in peach (plum subfamily) fruit, in which linalool is a key odorant that affects fruit aroma and consumer preference; over-expression of *terpene synthase gene 3* led to linalool accumulation [2]. Thus, small changes in volatile content have the potential to affect fruit flavor quality, there is an emphasized interest to regulate fruit flavor related volatiles, and epigenetic regulation of terpenoids is also a control strategy during fruit ripening [6,7]. To facilitate the breeding of Rosaceae species with desirable sensory qualities and improve their qualities under stress conditions, a better understanding of the genetic determinants of aroma and flavor in general and terpenes in particular is required.

Terpenoids represent the largest group of natural products and make up diverse secondary metabolites. Terpenoid composition not only represents a critical attribute in determining the quality of horticultural food products, such as taste and aroma, but also functions widely in plant development and defense, such as attracting pollinators, defending against herbivores, and acting as anti-bacterial agents [8,9,10]. Plant terpenoids (isoprene-C5, monoterpenes-C10, sesquiterpenes-C15, diterpenes-C20, and polyterpenoids-C5xn) are some compounds derived from isomeric 5-carbon building blocks isopentenyl diphosphate (IPP) and dimethylallyl diphosphate (DMAPP) [11,12]. About 50,000 terpenoid metabolites including monoterpenes, sesquiterpenes, and diterpenes have been identified in higher plants, liverworts, and fungi [11,12]. In general, for a better adaptation to a local ecological niche, each species typically synthesizes only a small fraction of terpenoid metabolites [9,13]. In plants, geranyl diphosphate (GPP), farnesyl diphosphate (FPP), and geranylgeranyl diphosphate (GGPP) are the precursors for monoterpenes, sesquiterpenes, and diterpenes, respectively; terpene synthase genes (TPSs) are responsible for converting them into a multitude of cyclic and acyclic terpenoids [12]. The characteristic catalytic function of TPSs is to generate multiple terpenoid products using one substrate, thus collectively contributing to numerous different structures of plant terpenoids in addition to modifying enzymes [12]. Generally, the TPS family is characterized by two large domains including the N-terminal domain (PF01397) and the C-terminal metal cofactor binding domain (PF03936) [14]. The N-terminal domain possesses a conserved RRX8W (R, arginine, W, tryptophan, and X, alternative amino acid) motif and the C-terminal domain contains two highly conserved aspartate-rich motifs, the DDxxD motif and the NSE/DTE motif. The DDxxD motif is involved in the coordination of divalent ion(s), water molecules, and the stabilization of the active site, and is found among all functional TPSs [3]. The NSE/DTE motif flanks the entrance of the active site and function in binding a trinuclear magnesium cluster [14,15]. TPSs are split into seven subgroups based on their amino acid sequence relatedness, namely TPS a-g. The majority of TPSs in most plants fall into one or two clades and the TPS-d clade was only encoded in gymnosperms [15,16]. Different TPS clades differ considerably in catalysate and sequence. As the largest clade, the TPS-a clade mainly encodes sesquiterpenes. TPS-b and TPS-g clades are clustered closely to TPS-a; the TPS-b clade contains the conserved R(R)X8W motif and usually encodes monoterpenes, while the TPS-g clade lacks the conserved R(R)X8W motif, and functions in producing mono- and sesquiterpenes [17]. The TPS-c clade is characterized by the “DXDD” motif but not the “DDXXD” that was detected in other clades, and mainly functions in producing diterpene products [17]. The TPS-d clade is only encoded in gymnosperms, and function in producing mono- and sesquiterpene products. The TPS-e/f clades are clustered closely, and mainly encode diterpene products [17]. TPS-a/b clades lack the N-terminal γ domain characteristic of diterpene synthases found in clades c, e, and f. In general, the biosynthesis of isoprene, monoterpenes, and diterpenes occurs in the plastid and the biosynthesis of sesquiterpenes occurs in the cytosol [17].

So far, the TPS gene family members have been characterized in many plant species. The sizes of TPS families in the majority of sequenced plants genomes range from 1 to 100. The TPS families probably evolved through duplication of genes followed by functional divergence [13]. The bryophyte *Physcomitrella patens* has a single TPS gene [13]. In *Arabidopsis thaliana*, *Vitis vinifera*, *Ocimum sanctum*, *Daucus carota*, tomato, and *Camellia sinensis*, 32, 69, 81, 19, 44, and 80 TPSs have been identified, respectively [13,18,19,20]. However, not all of the TPSs were functional. In recent years, the genomes of Rosaceae species such as peach, plum, apple, pear, and strawberry have been sequenced, which has significantly promoted the related studies of this family. Although abundant terpenes were characterized in different tissues of Rosaceae species [3,4,21], a comprehensive study on TPSs has not been reported in Rosaceae.

In this study, we mainly focused on three subfamilies in Rosaceae that are of economic importance as food crops, and did not select the *Spirea* subfamily, as the *Spirea* subfamily is only composed of ornamental flowers; thus, we excluded it from this study. For each subfamily, we chose two representative species with available sequenced genomes of high quality (chromosome scale). Based on the annotated genomes, we carried out the identification, characterization, and metabolite pathway mapping predictions of all TPSs encoded by eight Rosaceae species, including three Prunoideae species, three Maloideae species, and two Rosoideae species. We classified these TPSs into putative TPS-like proteins containing either PF01397 or PF03936 and complete ones containing both of them, respectively. The analysis of phylogeny, gene structure, and expression patterns were conducted with a special focus on their family number distribution and tissue expression patterns among subfamilies and different TPS clades. The findings revealed the diversity and functional evolution of TPSs in Rosaceae. The results provide a foundation for the exploration of TPSs to improve the understanding of the evolution and biosynthesis of terpenoids in Rosaceae.

## 2. Results

### 2.1. Identification of TPS Family Members in Rosaceae

To explore the distribution of TPSs among Rosaceae species and the evolutionary trajectory in the subfamily, we selected eight Rosaceae species for the identification of TPSs, including three Prunoideae species (*P. persica*, *P. mira*, *P. mume*), three Maloideae species (*P. betulifolia*, *M. domestica*, *M. baccata*) and two Rosoideae species (*F. vesca*, *R. chinensis*) (Table 1). BLASTP and HMM searches were performed against their entire protein sequences, and these two approaches produced the similar number of hits, indicating the relative conservation of the TPS family. We merged the hits together and verified them for the existence of Pfam domains PF03936 (metal-binding domain) and PF01397 (N-terminal TPS domain). Pfam domain distribution in the TPSs of eight Rosaceae species is listed in Table S1. As a result, hundreds of complete TPSs that contained both domains were identified. A recent study used this similar approach to detect TPSs in peach [2], and it detected 38 full-length TPSs with both domains; another study detected cultivated apple contained 55 putative TPS genes [4], the similar amount of TPSs as our results indicates the approach used in our study is reliable. For each Rosoideae species, we found that a certain ratio (64.29–0%) of putative TPSs was composed of both domains. The reference information and family number distribution of TPSs is listed in Table 1. The average family number of all TPSs in the Rosoideae species is the highest (65–76), followed by Maloideae (48–56) and Prunoideae (10–45). The Prunoideae species showed varied numbers of TPSs; there were only 10 putative TPSs identified in *P. mira*. In contrast, 30 and 45 putative TPSs were detected in *P. mume* and *P. persica*, respectively, whereas for the complete TPSs with both domains, 38, 36, and 43 TPSs were identified in *P. persica*, *M. domestica*, and *F. vesca*, respectively. *M. domestica* displayed a lower ratio of complete TPSs, with less than 64.29%, while the ratio in Prunoideae was up to 90%. All the putative TPSs were renamed numerically with the abbreviation of species names as a prefix (Table S2); only complete TPSs that contained both domains were used for the subsequent analysis.

### 2.2. TPS Classification and Motif/Domain Annotation

**Figure 1:**
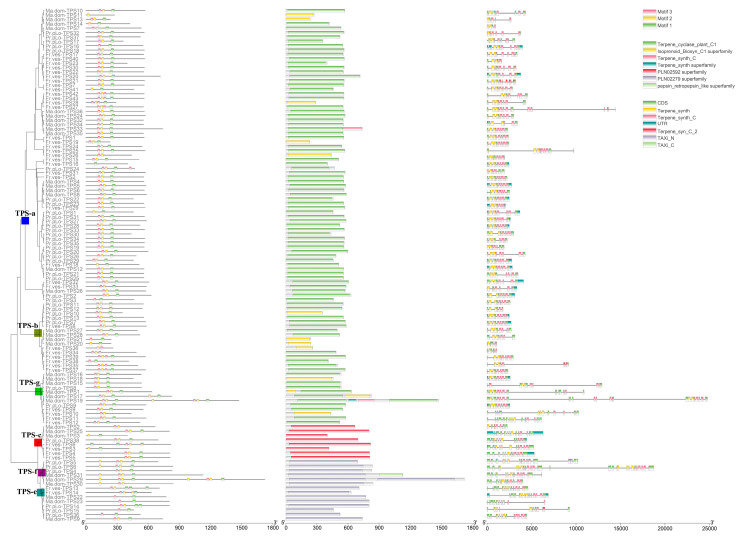
Phylogenetic relationship and distribution of motif/domain of TPSs in three Rosaceae species (*P. persica*, *M. domestica*, *F. vesca*). The phylogenetic tree is shown on the left panel, while conserved motifs, conserved domains, and Pfam domains are shown on the right three panels. The phylogenetic tree from full-length amino acid sequences was constructed using the MEGA with maximum likelihood (ML) method. The conserved motifs were assessed using the online MEME software. The conserved domain was annotated based on the conserved domain database in NCBI, whereas the gene structure and domains were annotated by using the PfamScan tool. The conserved motifs and domains are shaded in different colors. The root nodes of TPS-a, TPS-g, TPS-b, TPS-c, TPS-e, and TPS-f clades are indicated by blue, green, yellow-green, red, benzo, and purple, respectively.

**Figure 2:**
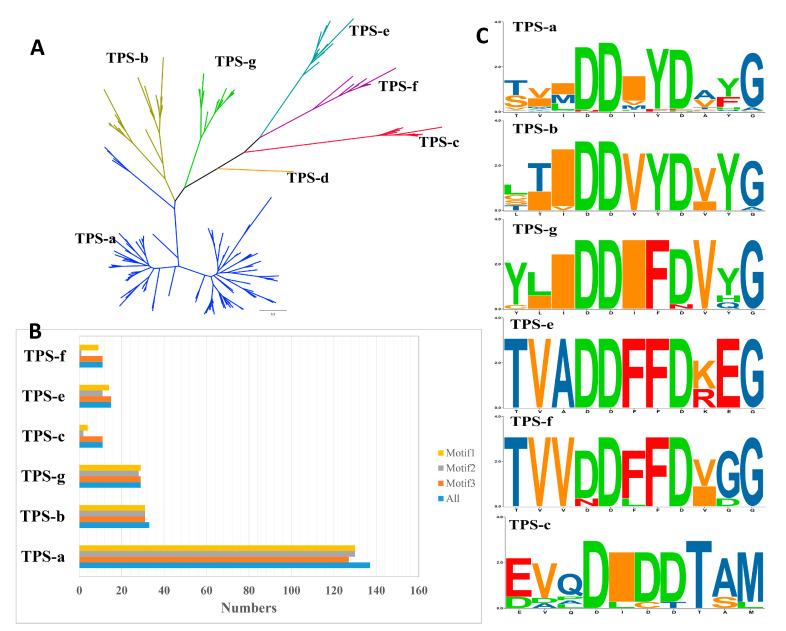
The unrooted phylogenetic tree of TPSs and motifs comparison between different TPS clades. (**A**) The maximum-likelihood phylogenetic tree of the TPS proteins in three Rosaceae species (*P. persica*, *M. domestica*, *F. vesca*). The TPS-a, TPS-g, TPS-b, TPS-c, TPS-e, and TPS-f clades are shaded in blue, green, yellow-green, red, benzo, purple, respectively. (**B**) The frequency of different motifs among different TPS clades. (**C**) The seqLogo of ‘DDxxD’ motif in the C-terminal domain of different TPS clades, the bit score represents the information content for each position in the sequence.

All the TPSs containing both PF01397 and PF03936 domains were subjected to classification and motif annotation. TPSs from the three representative Rosaceae species (*P. persica*, *M. domestica*, and *F. vesca*) were chosen for the visualization of the classification and domain/motif distribution in TPSs. After removing putative TPSs lacking both domains, a total of 117 TPSs in the three species were used for phylogenetic construction (Figure 1 and Figure S1). The phylogenetic topology revealed that all the TPSs were divided into seven known clades TPS a-g. TPS-b and TPS-g clustered with the TPS-a clade and forms a large branch. TPS-e and TPS-f formed sister clades and clustered close to the TPS-c clade. No TPSs clustered with the TPS-d clade, which was only encoded in gymnosperms. The conserved motifs were constructed using the online MEME software and three conserved motifs (motifs 1, 2, 3) were detected in nearly all the TPSs (109, 96, and 102). However, the frequency and distribution of these motifs varied among TPSs. For example, *Ma.dom-TPS11* only contained motif 2, and lost motifs 1 and 3, whereas *Fr.ves-TPS28* contains an extra copy of motif 3. The motif distribution of TPSs in the eight Rosaceae species is listed in Table S1; the significant E-value indicates the reliability of the identified motifs. For different clades, we found that the motif composition of TPS-a/b/g clades is more conservative than that of TPS-c/e/f, as shown in Figure 2B; nearly all TPSs in these clades contain all of the three motifs. In contrast, many TPSs from TPS-c/e/f lost motif 2, indicating the differences of motif distribution among clades. The conserved domain (CD) annotation used by the CD-search tool in NCBI revealed the discrepancy of domain annotation among different clades. CD domains Terpene_cyclase_plant_C1 (accession: cd00684) and Isoprenoid_Biosyn_C1 (accession: cd00385), which both belong to superfamily Isoprenoid_Biosyn_C1 superfamily (accession: cl00210), were annotated in clades of TPS-a, TPS-b, and TPS-g. PLN02279 super family (ent-kaur-16-ene synthase) was annotated in TPS-e/f clades. PLN02592 superfamily (ent-copalyl diphosphate synthase) was annotated in TPS-c clade. Pfam domain annotation results verified that each full-length TPS is characterized by two conserved domains with PF01397 (N-terminal) and PF03936 (C-terminal). The protein lengths of TPSs ranged from 232 AA to 1726 AA, showing a wide distribution of TPS lengths. One of the conserved aspartate-rich motifs in the C-terminal domain that is involved in the coordination of divalent ions, water molecules, and the stabilization of the active site was characterized based on motif sequences alignment (Figure 2C). The conservation of amino acid composition varied among different TPS clades. The TPS-c subfamily is characterized by the “DXDD” motif but not the “DDXXD” motif that was detected in other clades.

### 2.3. Varied Family Number of TPSs among Different Clades in Rosaceae

**Figure 3:**
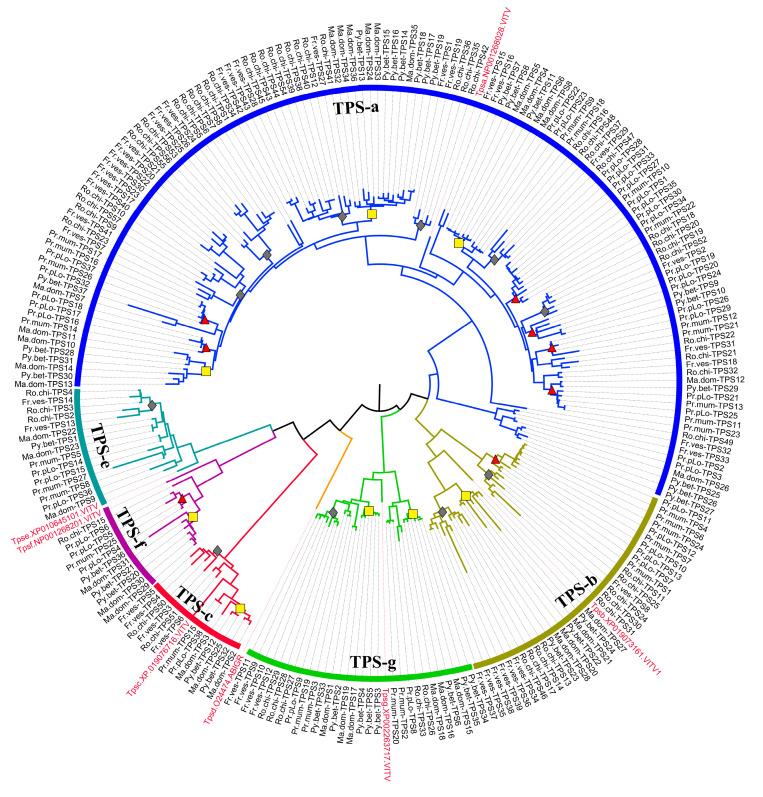
Phylogenetic tree of complete TPSs from six representative Rosaceae species. The TPS members were identified from six species including two Prunoideae species (*P. persica*, *P. mume*), two Maloideae species (*M. domestica*, *P. betulifolia*), and two Rosoideae species (*F. vesca*, *R. chinensis*); only those TPSs containing both PF01397 and PF03936 domains were used. The phylogenetic tree from full-length amino acid sequences was constructed using the MEGA with maximum likelihood (ML) method. Representative sequences of TPSs from *Vitis vinifera* were used as outgroups. The branches of TPS-a, TPS-g, TPS-b, TPS-c, TPS-e, and TPS-f clades are indicated by blue, green, yellow-green, red, benzo, and purple colors, respectively. The lineage-specific expansion of TPSs in Prunoideae, Maloideae, Rosoideae are indicated by yellow squares, grey diamonds, red triangles, respectively. The enlarged phylogenetic tree with bootstrap values is shown in Supplementary Figure S2.

To explore the evolution of TPSs in Rosaceae, we chose two representative species from each subfamily in Rosaceae for phylogenetic tree construction, including two Prunoideae species (*P. persica*, *P. mume*), two Maloideae species (*M. domestica*, *P. betulifolia*), and two Rosoideae species (*F. vesca*, *R. chinensis*). The detailed distribution of family numbers of different TPS clades is listed in Table 2. The TPS-a clade is the major determinant of family size of individual species; it is the largest group with more TPS copies, followed by TPS-b and TPS-g, TPS-c/e/f have a relatively small family size. On the whole, Rosoideae species owned more TPS copies, followed by Maloideae species, and Prunoideae species. The family number of TPSs from *R. chinensis* is up to 57, of which 37 are TPS-a members. The six Rosaceae species that we chose nearly contained all five TPS clades; however, no TPS-f member was detected in *F. vesca.* The family number distribution of different TPS clades also varied among three Rosaceae subfamilies. For example, more than two TPS-c gene copies existed in the Maloideae and Rosoideae subfamilies, while only one copy was detected in Prunoideae species. The diversity of family number of TPS-f is also obvious; both *M. domestica* and *P. betulifolia* owned three copies, while for Rosoideae species, only one copy was encoded in *R. chinensis.* For Prunoideae species, one copy of TPSs from the TPS-f clade was detected in *P. mira* and *P. mume*, while three copies were detected in *P. persica*, indicating the recent expansion of the TPS-f clade in modern peach. The phylogenetic relationship revealed that lineage-specific expansions of the TPSs were widely observed within different TPS clades. These expansions are denoted in the phylogenetic tree (Figure 3 and Figure S2), the clustering relationship indicated that many expansions occurred after the split of sister lineages, such as the split of Maloideae and Prunoideae. Two Rosoideae species (*F. vesca*, *R. chinensis*) owned more lineage-specific expansions compared with the other two Rosaceae subfamilies, especially in the branch of TPS-a clade. On the other hand, in the lineage-specific expansion branch, except that many copies were shared by sister species, species-specific expansions were also detected widely. *R. chinensis* was also the most striking species with more copies of TPSs arising by species-specific expansion.

### 2.4. Chromosomal Location, Synteny Analysis of TPS Family Members in Rosaceae

**Figure 4:**
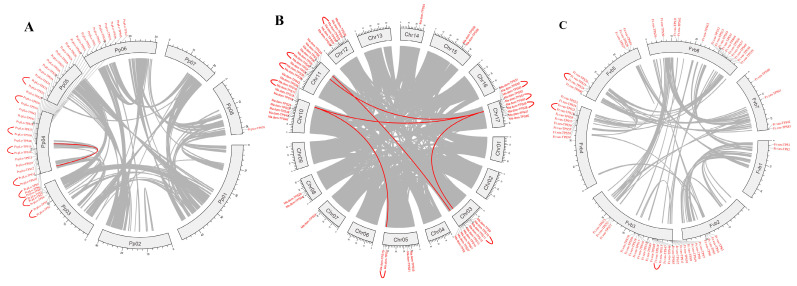
Inter-chromosomal relationships of TPSs in *P. persica* (**A**), *M. domestica* (**B**), and *F. vesca* (**C**). Grey lines in the circle indicate the collinear blocks in the *P. persica* or *M. domestica* genome. The red lines in the circle highlight the segmental duplicated TPS gene pairs, while the red curve outside the circle indicates the tandemly duplicated TPS gene pairs produced by MCSscanX.

**Figure 5:**
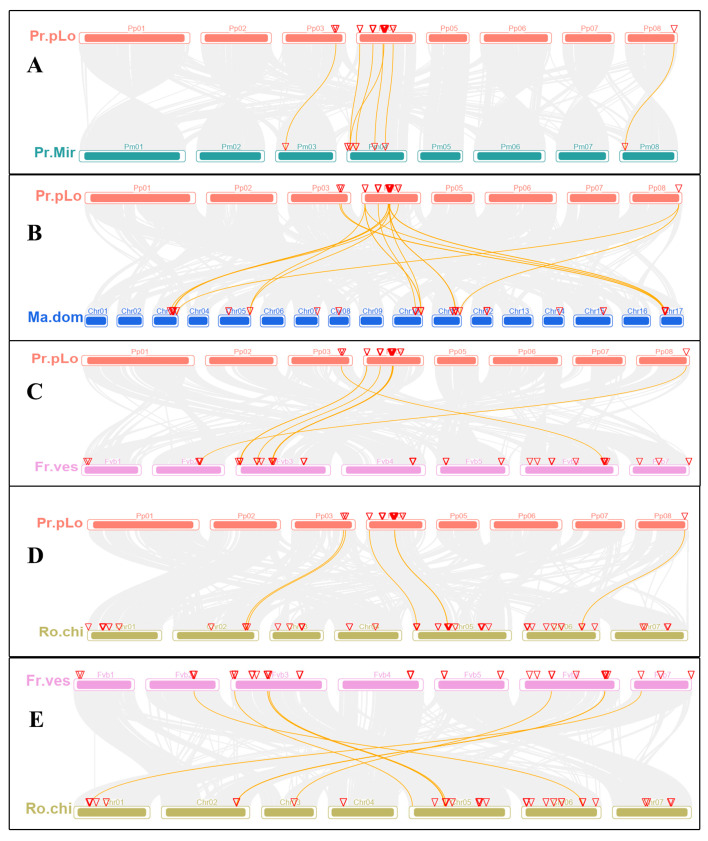
Synteny analysis of TPSs among Rosaceae species. Grey lines in the background indicate the collinear blocks between different genomes, while the yellow lines highlight the syntenic TPS gene pairs. The chromosome is indicated by different colored boxes and labeled by Pr.pLo (*P. persica*), Pr. Mir (*P. mira*), Ma.dom (*M. domestica*), Fr.ves (*F. vesca*), Ro.chi (*R. chinensis*). Collinear relationships between *P. persica*-*P. mira*, *P. persica*-*M. domestica*, *P. persica*-*F. vesca*, *P. persica*-*R. chinensis*, *F. vesca*-*R. chinensis* are shown in (**A**–**E**), respectively.

The chromosomal location maps of the TPSs in Rosaceae species were constructed by TBtools, as shown in Figures S3–S9, which indicates the diversity of distribution on chromosomes. For *P. persica*, TPSs were mainly distributed on three chromosomes (chromosomes 3, 4, and 8). Most TPSs (39) were distributed on the 4th chromosome, while five and one TPSs were located on the 3rd and 8th chromosomes, respectively. For the Rosoideae and Maloideae species, TPSs were widely distributed on the 8th and 10th chromosomes, respectively. On the whole, plants from the same subfamily shared a similar chromosome distribution of TPSs. From the physical location maps, we observed that the tandem arrays of TPSs are quite extensive, as in *P. persica*, about 30 TPSs cluster across a stretch of 1051 kb on the 4th chromosome, in *M. domestica*, six TPSs occur in a 200 kb stretch on 10th chromosome. These tandem arrays are likely the consequence of duplication by unequal crossover; genes in tandem arrays are typically highly homologous to each other. For *P. persica*, *M. domestica*, and *F. vesca*, synteny analysis within genomes was conducted to determine their duplication events, as shown in Figure 4, which indicated that genome segmental and tandem duplication were together the driving force for the expansion of the TPS gene family in Rosaceae. For *M. domestica* and *P. persica*, segmental and tandem duplication both contributed to family expansion; for *F. vesca*, tandem duplication played the most important role in the family expansion. MCScanX was used to identify possible collinear blocks between genomes in Rosaceae; the syntenic map between them was constructed, as shown in Figure 5, which showed plenty of syntenic relationships resulting from genome duplication and recombination.

### 2.5. Ka/Ks Ratios of TPS Family Members in Rosaceae

In addition, based on the phylogenetic relationship in Figure 3, we assumed TPSs pairs that derived from recent duplication as paralogs. There were two types of paralogs: one is the “between-species paralog” and the other is “within-species paralog” [22]. In this study, we only used the latter type of paralogs. As a result, a total of 82 TPS paralogs from recent duplication were found in the six Rosaceae species (*P. persica*, *P. mume*, *M. domestica*, *P. betulifolia*, *F. vesca*, *R. chinensis*). To explore the selection pressure in the evolution of TPSs, the Ka/Ks values were calculated for the six Rosaceae species (Table S3); a Ka/Ks value of less than one implies purifying selection, Ka/Ks = 1 represents neutral selection, and Ka/Ks > 1 indicates positive selection. The results showed that the Ka/Ks values of TPS paralogs were mostly less than one, suggesting that these genes evolved under purifying selection. There were five gene paralogs with Ka/Ks values greater than one, including three pairs of the TPS-a clade (Ro.chi-TPS18/Ro.chi-TPS20, Ma.dom-TPS14/Ma.dom-TPS13, and Ma.dom-TPS6/Ma.dom-TPS8), one pair from the TPS-b clade (Fr.ves-TPS11/Fr.ves-TPS9), and one pair from the TPS-c clade (Ma.dom-TPS29/Ma.dom-TPS30), which indicates that they were evolved under positive selection. Based on Ks values, the divergence time was calculated, which showed that five gene paralogs were diverged less than 5.5 Mya, especially for Ro.chi-TPS18 and Ro.chi-TPS20, and their divergence time was estimated to be around 0.55 Mya, indicating these gene paralogs were diverged recently. We observed that the divergence time varied among different clades. The divergence time of paralogs from TPS-e were all more than 11.4 Mya, which is higher than other clades, indicating the TPS-e clade diverged relatively anciently.

### 2.6. Function Diversity of TPSs in Rosaceae

For the six representative plants shown in Figure 3, we predicted their function based on blast searching against Uniprot and KEGG pathway databases; their subcellular localization was also predicted using TargetP [23] and pLoc-mPlant (www.jci-bioinfo.cn/pLoc-mPlant/, accessed on 24 August 2021). Detailed information is listed in Table 3 and Table S2. Subcellular localization analysis indicated that TPSs were substantially localized to cytoplasm and chloroplasts, and only a few TPSs were localized to mitochondria. TPSs from the TPS-a clade were mostly located in cytoplasm and chloroplasts, and demonstrated varied function diversity. Most of the characterized TPSs (~95%) in the TPS-a clade are involved in sesquiterpenoid and triterpenoid biosynthesis, and monoterpene biosynthesis pathway, using geranyl diphosphate GPP and farnesyl diphosphate (FPP) as substrate. Whereas for *M.* *domestica* and *P. betulifolia*, several TPSs from TPS-a clade are involved in diterpenoid biosynthesis that used geranylgeranyl diphosphate (GGPP) as substrate. The finding suggested both the cytosolic mevalonic acid (MVA) pathway and the plastidic methylerythritol phosphate (MEP) pathway coexist in the TPS-a clade. All characterized TPSs in TPS-b clade are involved in either a sesquiterpenoid or monoterpene biosynthesis pathway. The members from the TPS-g clade mainly function in producing acyclic mono- and sesquiterpenoid products. TPS-c and TPS-e clades mainly participate in diterpenoid biosynthesis. Surprisingly, despite the close relationship between TPS-f and TPS-e/c clades, we found that TPS-f members are involved in sesquiterpenoid and triterpenoid biosynthesis, and monoterpene biosynthesis pathway. Furthermore, we observed that TPSs from the same clade participated in different pathways, such as, for TPS-e clade, TPSs were all predicted as ent-kaur-16-ene synthases with exceptions in the *M. domestica* that involved in other pathways. For the Prunoideae subfamily, the function of TPSs among different clades was relatively conserved in three plants, and more TPS copies from the TPS-a clade involved in the monoterpene biosynthesis pathway were detected in *P. persica*. However, the functional diversity of TPSs is more obvious for Maloideae and Rosoideae species, such as the TPS-a clade in *P. betulifolia*. The above findings indicated that the TPS family in Rosaceae species possesses remarkable flexibility to evolve enzymes substrate specificity, and different clades expand in different lineages by gene duplication and divergence. It can be expected that proteins with altered subcellular localization and new substrate specificities would have evolved.

### 2.7. Tissue-Specific Expression of TPSs in Rosaceae

**Figure 6:**
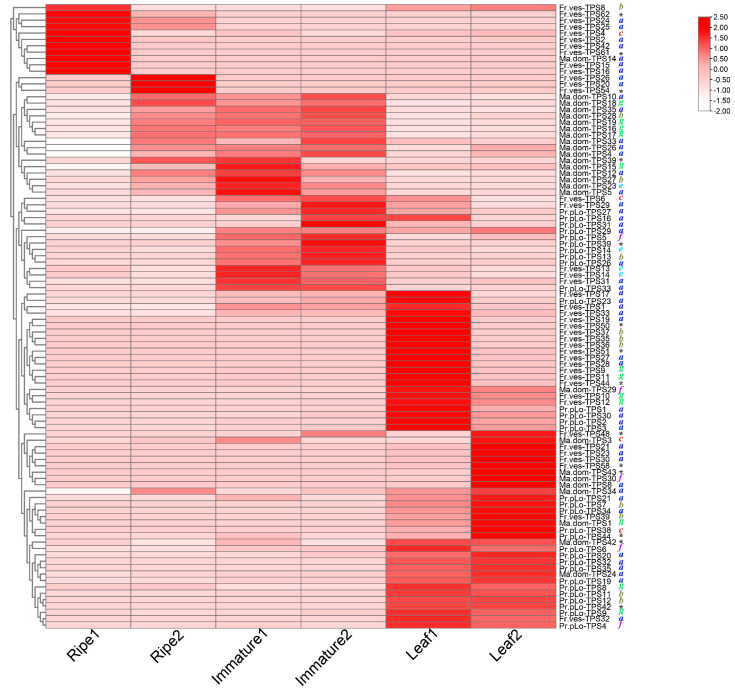
Expression pattern of expressed TPSs in the three Rosaceae species (*P. persica*, *M. domestica*, *F. vesca*). Heat mapping of TPSs gene expression in three Rosaceae species. The x-axis represents different samples (ripe fruit, immature fruit, and leaf), the y-axis represents TPSs. There are two replicates for each tissue. The rows and columns were clustered based on row-scale normalized expression values. TPSs clades were shown on the right by different colors; putative TPSs without both domains are indicated by black stars.

**Figure 7:**
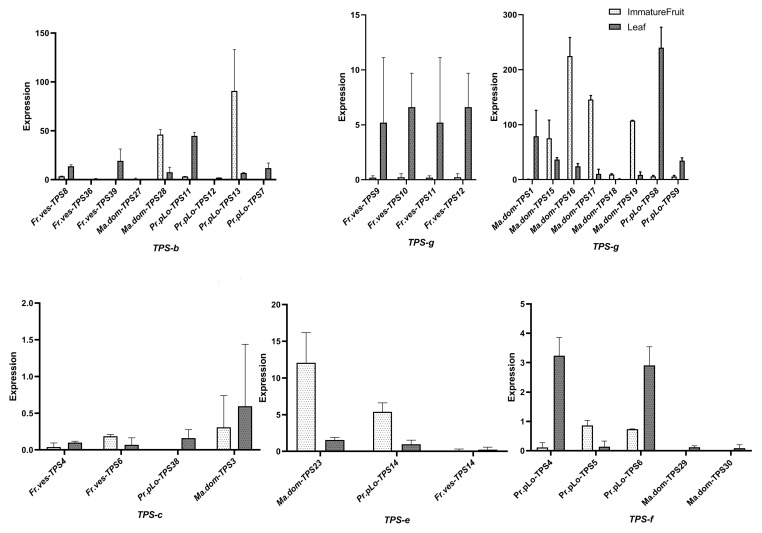
The expression level of TPSs from clades b, c, g, e, and f in the three Rosaceae species (*P. persica*, *M. domestica*, *F. vesca*). Tissues (immature fruit, leaf) are indicated by different colors.

For the three representative Rosaceae species (*P. persica*, *M. domestica*, and *F. vesca*), based on RNA-seq data of different tissue development (ripe fruit, immature fruit, leaf), each tissue consisted of two replicates. We examined the tissue expression profiles of TPSs, as listed in Table S4, and observed that TPSs in the three Rosaceae species demonstrated tissue-specific expression (see Figure 6 and Figure S10). There were about 31, 26, and 41 TPSs expressed in at least one tissue for *P. persica*, *M. domestica*, *F. vesca*, respectively; surprisingly, 11 of them were putative TPSs without both domains, such as *Fr.ves-TPS61*, *Fr.ves-TP2*, *Pr.pLo-TPS42*, *Ma.dom-TPS42*, etc. Whereas many TPSs with both domains were not expressed in any of the three tissues, these findings indicated the complexity of TPSs expression. In ripe fruits, expressed TPSs were only detected in strawberries; for *M. domestica* and *P. persica*, no TPSs were expressed in ripe fruits. In addition, we observed that most TPSs were exclusively or highly expressed in one tissue, as shown in Figure 7, such as *Pr.pLo-TPS13*, *Ma.dom-TPS28*, and *Fr.ves-TPS9* genes; they were highly or merely expressed in immature fruit or leaf. We further examined the expression pattern between different TPS clades; for the three plants, different TPSs from TPS-a clade were widely distributed in different tissues, indicating the functional diversity of TPS-a clades. However, the expression from other TPS clades varied among species, such as TPS-b clade; its members in *M. domestica*, *Ma.dom-TPS28*, were only highly expressed in immature fruit, but its counterparts in *P. persica*, *Fr.ves-TPS11 and Fr.ves-TPS13*, displayed an opposite expression pattern despite their high homology. *Fr.ves-TPS11* was highly expressed in leaf, while *Fr.ves-TPS13* was highly expressed in immature fruit, indicating the function differentiation after the recent duplication. Similarly, for TPS-g clade, its members (*Fr.ves-TPS9-12*) in *F. vesca* and members (*Pr.pLo-TPS9*, *Pr.pLo-TPS9*) in *P. persica* were only highly expressed in leaf, while the counterparts in *M. domestica* (*Ma.dom-TPS1*, *Ma.dom-TPS15-18*) showed different tissue expression specificity. In addition, we found that the TPS-f clade showed a larger variation between three species. *F. vesca* lost the TPS-f genes, *M. domestica* had two copies that displayed a low expression level, while *P. persica* owned three copies, which is higher than all other species. Two copies were relatively highly expressed in leaf, and one copy was expressed in immature fruit. They were predicted as s-linalool synthases; their product s-linalool is assumed to be an important flavor component in *P. persica* [2]. On the whole, the above finding indicated that TPSs in Rosaceae also underwent extensive expression differentiation after the split of sister lineages and gene duplication.

## 3. Discussion

In the plant kingdom, terpenes are traditionally classified as secondary metabolites. Thousands of terpenes have been found, and have proven to play significant roles not only in resistance against stress conditions but also in flavor formation [19,20,24,25]. However, each species is capable of synthesizing only a small fraction, whose synthesis has evolved in plants as a result of selection for increased fitness via better adaptation to the local ecological niche of each species [12]. Terpene synthases are responsible for the synthesis of the various terpene molecules [12]; plant TPS gene families are a medium-sized group, and display varied numbers of TPS families among different species [16]. The Rosaceae family has significant economic value, including the fruit crops and ornamental flowers; however, comprehensive molecular evolutionary and function analysis of TPSs remain elusive. In this study, we screened for the TPS family from eight Rosaceae species. We identified TPSs by detecting both domains and either single domain separately, thus minimizing the chance of missing putative TPSs. We found this family in Rosaceae is a midsized family, as identified in a previous study [13], ranging from 10 TPSs in *P. mira* to 76 in *R. chinensis*. Domain loss for either N-terminal or C-terminal occurred frequently in Rosaceae species.

All the Rosaceae TPSs in this study were divided into seven known clades, TPS a–g. The family numbers of different TPS clades varied among three Rosaceae subfamilies; for example, more than two TPS-c gene copies existed in Maloideae and Rosoideae subfamilies, while only one copy was detected in Prunoideae species. The average number of TPSs in Prunoideae species is lower than that of Maloideae and Rosoideae; no recent WGD except a triplicated arrangement could limit the expansion of TPSs in Prunoideae. Additionally, fewer TPSs in the early Prunoideae species *P. mira*, but more TPSs in modern peach *P. persica*, further revealed the evident evolutionary plasticity of the TPS family. Lineage- and species-specific expansions of the TPSs were widely observed within different TPS clades in Rosaceae. The varied family number and differentiation of TPSs in Rosaceae may play roles in the specialization of essential traits and species differentiation. It has been proposed that lineage-specific genes have a greater chance to contribute to phenotypic variations because their roles are not essential. Further synteny analysis showed that segmental and tandem duplications were both the driving force for the expansion of the TPS gene family in Rosaceae; for *M. domestica* and *P. persica*, segmental and tandem duplication contributed to family expansion; and for *F. vesca*, tandem duplication played the most important role in the family expansion. Ka/Ks calculations further revealed that TPSs genes mainly evolved under purifying selection, except for several pairs; the divergent time indicated TPS-e clade was diverged relatively anciently.

Since closely related enzymes differ in their product profiles, subcellular localization, or substrates, the prediction strategy based on sequence similarity usually cannot accurately describe the specialized function of TPSs, and only roughly obtained their involved pathways. In our study, we still predicted their function based on blast searching against Uniprot and KEGG pathway databases, the results revealed the functional diversity between different TPS clades and species. For the three Prunoideae species, the functional classification of TPSs among different clades was relatively conservative except for the family number variation. Whereas for the other two subfamilies, the putative function of TPSs demonstrated wide diversity, such as TPS-a clade in *P. betulifolia*, up to seven different types of synthases were predicted. In addition, we observed that early diverged TPS-e clade is conserved in function and most of their members were all predicted as ent-kaur-16-ene synthases with exceptions in *M. domestica*. Despite TPS-e and TPS-f being sister clades that clustered together, we found that their function had undergone differentiation, in contrast to TPS-e that mainly participated in diterpenoid biosynthesis, most TPS-f members are involved in the monoterpene biosynthesis pathway. The expansion of TPS-f in *P. persica* and their product, S-linalool synthase, is the essential aromatic substance in peach fruits [21]. Our findings indicated that the TPS family in Rosaceae species possesses remarkable function diversity; different clades expand in different lineages by gene duplication and divergence. The generation of altered subcellular localization, and new substrate specificities of TPSs, is a dynamic process that specialized the trait differentiation. However, experimental data like metabonomics, enzyme assays, are also needed to verify these observations.

The expression profiling results on the *TPS* gene family in three Rosaceae species (*P. persica*, *M. domestica*, and *F. vesca*) showed that most identified TPSs were expressed in at least one tissue. Most of the TPSs were specifically expressed in one certain tissue; the expressed TPSs in ripe fruits are rare. For each TPS clade, the expression pattern also varied among species, such as TPS-f genes, which demonstrated a high expression in *P. persica*, but a lower expression in *M. domestica*. We also found that many paralogs exhibited divergent expression patterns either in tissues or expression abundance, suggesting that expression divergence might significantly contribute to gene survival and function differentiation after gene expansion. It was worth noting that among the expressed TPSs, certain putative TPSs did not have both domains. In contrast, many TPSs with both domains were not expressed, and the finding suggested that in the complexity of TPSs expression, not all of the complete TPSs were functional, and some functional ones may lose activity in either one domain. These putative TPSs without both domains were assumed to be triggered by partial duplication and assumed to be pseudogenes for the loss of original function. A previous study found that a total of 12% of the pseudogenes still contained detectable open reading frames and were effectively expressed. The generated transcripts may contribute to the synthesis of small interfering RNA species that regulate parent transcripts [26]. Fast-evolving families involved in ubiquitination and secondary metabolism families always contain the highest number of pseudogenes [26,27]. Hence, the functionality of the expressed “fragmental” TPSs in our study still needs further investigation.

## 4. Material and Methods

### 4.1. Identification of TPSs in Rosaceae

A total of eight Rosaceae species are included in the identification of TPSs, namely three Prunoideae, three Maloideae, and two Rosoideae species. Their genomes have been completely sequenced and annotated. The genome files of the Rosaceae species were mostly downloaded from the NCBI (https://www.ncbi.nlm.nih.gov, accessed on 2 August 2021) and GDR (https://www.Rosaceae.org, accessed on 2 August 2021), the versions of genomes were all the recently released and chromosome-scale, detailed genome information was summarized in Table 1. Six representative sequences of TPS-a, TPS-b, TPS-c, TPS-e, TPS-f, and TPS-g from *Vitis vinifera* from a previous study [28], and one representative sequence of TPS-d from *Abies grandis*, were used as queries to search the corresponding subject protein sequences of each Rosaceae species. Two different methods were used to identify TPSs in Rosaceae species. First, we implemented BLASTP searches of the complete genome with an E-value cut-off of 0.00001 to reduce false positives, and the second method was Hidden Markove Model (HMM) profiles of TPS domains in these Rosaceae species by using HMMER software with an E-value cut off of 0.001 [29]. The redundant sequences were removed by manual inspection. Subsequently, we verified all sequences by checking the existence of Pfam domains PF03936 (metal-binding domain) and PF01397 (N-terminal TPS domain) using PfamScan tools with default parameters [30], Pfam-A was used as the searching database. PfamScan tools search the whole sequences against the Pfam database, and annotate the sequence blocks as known domains, only the significant domains are retained, it can simultaneously predicted the different domains in one protein. Ultimately, genes containing at least one TPS domain were confirmed as members of the TPS gene family and named in numerical order.

### 4.2. Motif Annotation, Subcellular Localization, and Physical Localization

The conserved motifs were predicted using the online MEME software with the following settings: the motif discovery mode was classic, site distribution was zero or one occurrence per sequence (zoops), the background was a 0-order background model, the maximum number of different motifs was 20, minimum motif width was 6, and maximum motif width was 50 [31]. A shuffling was also performed prior to the MEME/MAST analysis to validate the identified motifs. The conserved domain was annotated based on the conserved domain database (CDD v3.19) in NCBI. TargetP and pLoc-mPlant (www.jci-bioinfo.cn/pLoc-mPlant/, accessed on 24 August 2021) were used to predict the subcellular localization of TPS proteins [32]. For each species, we got the information of the TPSs on the corresponding chromosome according to the annotation documents and drew a sketch map of the gene’s physical location using TBTools [33]. Protein functions were also predicted based on blast searching against Uniprot and KEGG pathway databases under default parameters.

### 4.3. Phylogenetic Tree Construction and TPS Classification

The full-length sequences of TPSs protein sequence with both domains from Rosaceae species were used to perform sequence alignment and phylogenetic tree construction using MEGA7 program [34]. The ClustalW method was used for sequence alignment under default parameters, the Maximum Likelihood and Neighbor-joining methods based on the Jones-Taylor-Thornton (JTT) matrix-based model were used for phylogenetic tree construction, a Bootstrap method was used for the phylogeny test with 500 replications, rates among sites was Gamma distributed (G), and Partial deletion was used for gaps/missing data treatment, with site coverage cutoff at 90%. The produced tree was further embellished by the FigTree program (http://tree.bio.ed.ac.uk/, accessed on 2 September 2021). TPS members were classified by their clustering relation with the query sequence mentioned above. For each TPS clade, their TPS member’s sequences were further aligned by ClustalW method, and a sequence logo for the visualization of the highly conserved aspartate-rich motif in C-terminal domain was produced by TBTools [33].

### 4.4. Ka/Ks Analysis of TPS Family Members in Rosaceae

We chose six representative Rosaceae species for selection pressure analysis, including *P. persica*, *P. mume*, *M. domestica*, *P. betulifolia*, *F. vesca*, and *R. chinensis*. For each species, its TPSs paralogs were determined based on their phylogenetic relationship. If they belong to the same clade and derive from a single gene that was duplicated recently, we assumed they were paralogs. Sometimes those paralogs that arose from a duplication after the speciation event are called “within-species” paralogs [22]. The CDS and protein sequences of each TPS pair were used to compute the Ka (non-synonymous rates) and Ks (synonymous rates) by Ka/Ks calculator (http://services.cbu.uib.no/tools/kaks, accessed on 2 Octomber 2021). The date (*T*) of the duplication events was estimated by the formula *T* = Ks/2λ, where λ represents the estimated clock-like rate of synonymous substitution; in dicots, it was 1.5 × 10^−8^ substitutions/synonymous site/year [35].

### 4.5. Synteny Analysis and Detection of Tandemly/Segmentally Duplicated TPSs

To identify the synteny of TPS family genes among species, we performed all-to-all BLASTP between the genome of *P. persica* and other four species *(P. mira*, *M. domestica*, *F. vesca*, *R. chinensis*). The collinearity analysis was also performed between *F. vesca* and *R. chinensis.* For the three representative species (*F. vesca*, *M. domestica*, *P. persica*), we also performed self-blast by comparing protein-coding genes against their genome using BLASTP with an E-value cut-off of 0.00001. All BLASTP hits were used as input for software MCScanX (Multiple Collinearity Scan toolkit) [36] to identify possible collinear blocks within and between genomes of different species. Based on the self-blast results, we detected the tandemly/segmentally duplicated TPSs for each species. In addition to the tandem duplication that was determined by MCScanX, paralogues that were either adjacent or separated by ≤5 genes along a chromosome were also assigned as tandem duplicates. If paralogues were within known genomic duplication blocks, they were considered to be duplicated through segmental duplication. All intra/inter-genomic synteny relationships were visualized with TBtools [33].

### 4.6. Expression Analysis of TPSs in Rosaceae

For the three representative plants (*F. vesca*, *M. domestica*, *P. persica*), RNA-seq data of different tissues and development stages (ripe fruit, immature fruit, leaf) was retrieved from the fruitENCODE project [37]; each tissue consisted of two replicates. The fruitENCODE project aims to generate a comprehensive annotation of functional elements in seven climacteric fruit species (apple, banana, melon, papaya, peach, pear, and tomato) with sequenced reference genomes. The data were deposited in the SRA database of NCBI with accession number PRJNA381300. Transcriptome analysis was implemented by the protocol in a previous study [38]. The clean reads of RNA-seq data from each sample were mapped against the genome reference with HISAT2 [39]; each SAM file was converted into a BAM file, and sorted. Duplicates were removed with SAMtools [40,41]. Further transcript assembly and quantification of the read alignments were performed using Stringtie [42]. Gene expression levels were measured by FPKM (fragments per kilobase of transcript per million mapped reads) and normalized with the row-scale method. Heatmaps with all samples were plotted using the “HeatMap” function in TBtools.

## 5. Conclusions

In conclusion, this study investigated the TPS gene family in eight sequenced Rosaceae species through classification, chromosomal location, orthologous relationships, and duplication analysis. The distribution of the TPS gene family among Rosaceae species revealed a diversity of family number and function; lineage-specific expansion of the TPSs accompanied by frequent domain loss were widely observed within different TPS clades. We further provided their tissue-specific expression pattern in *F. vesca*, *M. domestica*, and *P. persica*, revealing the expression differentiation of TPSs between paralogs/species. The findings revealed the evolution of TPSs in Rosaceae and will be highly useful for further genetic improvement of Rosaceae species.

## Figures and Tables

**Table 1:** Summary of genome information and TPSs of sequenced Rosaceae species used in this study.

Subfamily	Species	Release Version *	The Prefix of Gene Symbol	Total Genes	Numbers of TPSs	Numbers of TPSs with Both Domains	Percent of TPSs with Both Domains	Numbers of Already Know TPSs
*Prunoideae*	*Prunus persica*	GDR, v2.0	Pr.pLo	26,873	45	38	84.44%	38 (both domains) [2]
	*Prunus mira*	GDR, v1.0	Pr.mir	26,958	10	9	90.00%	
	*Prunus mume*	NCBI, v1.0	Pr.mum	28,638	30	27	90.00%	
*Maloideae*	*Pyrus betulifolia*	GDR, v1.0	Py.bet	59,552	48	37	77.08%	
	*Malus x domestica*	GDR, HFTH1 v1.0	Ma.dom	44,677	56	36	64.29%	55 (all) [4]
	*Malus baccata*	CNGB, v1.0	Ma.bac	45,931	48	33	68.75%	
*Rosoideae*	*Fragaria vesca*	GDR, v4.0	Fr.ves	35,914	65	43	66.15%	
	*Rosa chinensis*	GDR, v1.0	Ro.chi	39,669	76	57	75.00%	

* GDR, Genome Database for Rosaceae; CNGB, China National GeneBank DataBase; NCBI, National Center for Biotechnology Information. Species used for the phylogenetic tree are highlighted in bold.

**Table 2:** Numbers of complete TPSs with both domains from Rosaceae species that were used for the phylogenetic tree of Figure 3.

TPSs Clade	TPS-a	TPS-b	TPS-g	TPS-c	TPS-d	TPS-e	TPS-f	Total
Motif	DDXYD	DDVYD	DDIFD	DIDDT	DXDD, DXXD	DDFFD	DDFFD	
*Prunus persica*	24	5	2	1	0	3	3	38
*Prunus mira*	0	0	4	1	0	3	1	9
*Prunus mume*	13	5	4	1	0	3	1	27
*Pyrus betulifolia*	20	3	8	2	0	1	3	37
*Malus x domestica*	17	4	6	3	0	3	3	36
*Rosa chinensis*	37	9	5	2	0	3	1	57
*Fragaria vesca*	26	7	4	4	0	2	0	43

**Table 3:** Putative functions of TPSs in six Rosaceae species.

TPSs Clade	TPS-a	TPS-b	TPS-g	TPS-c	TPS-e	TPS-f
Catalytic type	**a. monoTPS** 1, (−)-alpha-pinene synthase. C/P 2, S-linalool synthase **b. SesquiTPS** 1, (−)-germacrene D synthase. C/P 2, (3S, 6E)-nerolidol synthase. P 3, (E, E)-alpha-farnesene synthase. P **c. DiTPS** 1, Ent-kaur-16-ene synthase 2, Ent-copalyl diphosphate synthase. P 3, Copal-8-ol diphosphate hydratase. P	**a. monoTPS** 1, (−)-alpha-pinene synthase. C/P 3, Tricyclene synthase EBOS. C/P **b. SesquiTPS** 1, (−)-germacrene D synthase. C/P 2, (3S, 6E)-nerolidol synthase. P 3, (E, E)-alpha-farnesene synthase. P	**a. monoTPS** 1, (−)-alpha-pinene synthase. C/P 2, S-linalool synthase 3, Tricyclene synthase EBOS. C/P **b. SesquiTPS** 2, (3S, 6E)-nerolidol synthase. P **c. DiTPS** 2, Ent-copalyl diphosphate synthase. P	**a. monoTPS** 1, (−)-alpha-pinene synthase. C/P 3, Tricyclene synthase EBOS. C/P **b. SesquiTPS** 1, (−)-germacrene D synthase. C/P 2, (3S, 6E)-nerolidol synthase. P **c. DiTPS** 2, Ent-copalyl diphosphate synthase. P 3, Copal-8-ol diphosphate hydratase. P	**a. monoTPS** 1, (−)-alpha-pinene synthase. C/P **b. SesquiTPS** 2, (3S, 6E)-nerolidol synthase. P 3, (E, E)-alpha-farnesene synthase. P **c. DiTPS** 1, Ent-kaur-16-ene synthase	**a. monoTPS** 2, S-linalool synthase 3, Tricyclene synthase EBOS. C/P **b. SesquiTPS** 1, (−)-germacrene D synthase. C/P **c. DiTPS** 1, Ent-kaur-16-ene synthase
*Prunus persica*	a1 (19) *; b1 (5);	b3 (5);	b2 (2);	c2 (1);	c1 (3);	a2 (3);
*Prunus mume*	a1 (6); b1 (4);	b3 (5);	b2 (4);	c2 (1);	c1 (3);	a2 (1);
*Prunus mira*	b1 (1);	/	b2 (4);	c2 (1);	c1 (3);	a2 (1);
*Fragaria vesca*	a1 (8); b1 (18);	a3 (6); b3 (1);	b2 (4);	c2 (3), c3 (1);	c1 (2);	/
*Rosa chinensis*	a1 (10); b1 (16), b2 (5), b3 (5); c1 (1);	a1 (2), a3 (2); b1 (3), b3 (2);	a1 (3), a2 (1); a3 (1);	b1 (2);	c1 (3);	b1 (1);
*Pyrus betulifolia*	a1 (8), a2 (3); b1 (8), b2 (2), b3 (2); c2 (2), c3 (1);	b1 (3);	a1 (2), a3 (1); b2 (5),	a1(1), a3 (1);	c1 (1);	a3 (1);
*Malus x domestica*	a1 (4); b1 (4), b2 (2); c1 (5), c2 (2);	a3 (2); b2 (1), b3 (1);	a1 (1); b1 (3), b2 (1); c2 (1);	b1 (1), b2 (1); c2 (1);	a1 (1); b2 (1), b3 (1);	a3 (1); c1 (1);

Notes: *, a1 represents the catalytic type of TPS listed in the second row; number in the bracket “(19)” represents family number of this TPS in the corresponding species.

## Data Availability

All raw reads used in this work were deposited in NCBI Bio-Project with the accession number PRJNA381300.

## Supplementary Materials

The following supporting information can be downloaded at https://www.mdpi.com/article/10.3390/plants11060736/s1. Table S1: Pfam domain distribution in the TPSs of eight Rosaceae species (sheet a); motif distribution in the TPSs of three Rosaceae species (sheet b); the alternative splicing transcripts of putative TPSs in *F. vesca*, *M. domestica*, and *P. persica* (sheet c). Table S2: Information of all TPSs analyzed in this study, (XLSX); Table S3: Ka/Ks ratio of TPSs in Rosaceae; Table S4: Expression values of TPSs in *F. vesca*, *M. domestica*, and *P. persica*. Figure S1. A Neighbor-joining phylogenetic tree of TPSs of the TPS proteins in three Rosaceae species (*P. persica, M. domestica, F. vesca*). Figure S2. A maximum-likelihood phylogenetic tree of six representative Rosaceae species TPS gene family. The branches of TPS-a, TPS-g, TPS-b, TPS-c, TPS-e and TPS-f clades are colored in blue, yellow-green, green, red, purple and cyan, respectively. Figure S3. Chromosomal locations of TPS family members in peach ‘Lovell’ genome. The gene location visualize package of TBTools was used to exhibit chromosomal locations of the TPS genes. The number to the left of each chromosome represented the size of the chromosome in bp. Figure S4. Chromosomal locations of TPS family members in *Prunus mume* genome. The gene location visualize package of TBTools was used to exhibit chromosomal locations of the TPS genes. The number to the left of each chromosome represented the size of the chromosome in bp. Figure S5. Chromosomal locations of TPS family members in *Prunus mira* genome. The gene location visualize package of TBTools was used to exhibit chromosomal locations of the TPS genes. The number to the left of each chromosome represented the size of the chromosome in bp. Figure S6. Chromosomal locations of TPS family members in *Malus x domestica* genome. The gene location visualize package of TBTools was used to exhibit chromosomal locations of the TPS genes. The number to the left of each chromosome represented the size of the chromosome in bp. Figure S7. Chromosomal locations of TPS family members in *Pyrus betulifolia* genome. The gene location visualize package of TBTools was used to exhibit chromosomal locations of the TPS genes. The number to the left of each chromosome represented the size of the chromosome in bp. Figure S8. Chromosomal locations of TPS family members in *Fragaria vesca* genome. The gene location visualize package of TBTools was used to exhibit chromosomal locations of the TPS genes. The number to the left of each chromosome represented the size of the chromosome in bp. Figure S9. Chromosomal locations of TPS family members in *Rosa chinensis* genome. The gene location visualize package of TBTools was used to exhibit chromosomal locations of the TPS genes. The number to the left of each chromosome represented the size of the chromosome in bp. Figure S10. Expression pattern of TPS genes in three Rosaceae plants (*P. persica, M. domestica, F. vesca*). The x-axis represents different samples (ripe fruit, immature fruit and leaf), the y-axis represents TPS genes. There are two replicates for each tissue. The phylogenetic tree is shown on the left panel, the root nodes of TPS-a, TPS-g, TPS-b, TPS-c and TPS-e, TPS-f clades are indicated by blue, green, yellowgreen, red, benzo, and purple, respectively.

## Abbreviations

HMM	Hidden Markove Model
TPSs	terpene synthase genes
GPP	geranyl diphosphate
FPP	farnesyl diphosphate
GGPP	geranylgeranyl diphosphate
VOCs	volatile organic compounds
