# Supplementary material for: Diversity and Functional Evolution of Terpene Synthases in Rosaceae
Source: Plants (Basel). 2022 Mar 10;11(6):736. doi: 10.3390/plants11060736 (PMC8953233; doi:10.3390/plants11060736)
Supplement: Supplementary file 1 [file plants-11-00736-s001.zip › Supplementary.pdf]

Article

Diversity and Functional Evolution of Terpene Synthases in Rosaceae

Aidi Zhang [1, 2, *], Yuhong Xiong [1, 2, 3], Jing Fang [1, 2, 3], Xiaohan Jiang [1, 2, 3], Tengfei Wang [1, 2, 3], Kangchen Liu [1, 2, 3], Huixiang Peng [1, 2, 3], Xiujun Zhang [1, 2, *]

[1] Key Laboratory of Plant Germplasm Enhancement and Specialty Agriculture, Wuhan Botanical Garden, Chinese Academy of Sciences, Wuhan 430000, China

[2] Center of Economic Botany, Core Botanical Gardens, Chinese Academy of Sciences, Wuhan 430074, China

[3] University of Chinese Academy of Sciences, Beijing 100049, China

* Correspondence: zhangxj@wbgcas.cn

Wuhan Botanical Garden,

Chinese Academy of Sciences,

Wuhan 430072, China

Tel: +86-27-87700844

E-mail: zhangxj@wbgcas.cn

Supplementary Figures:

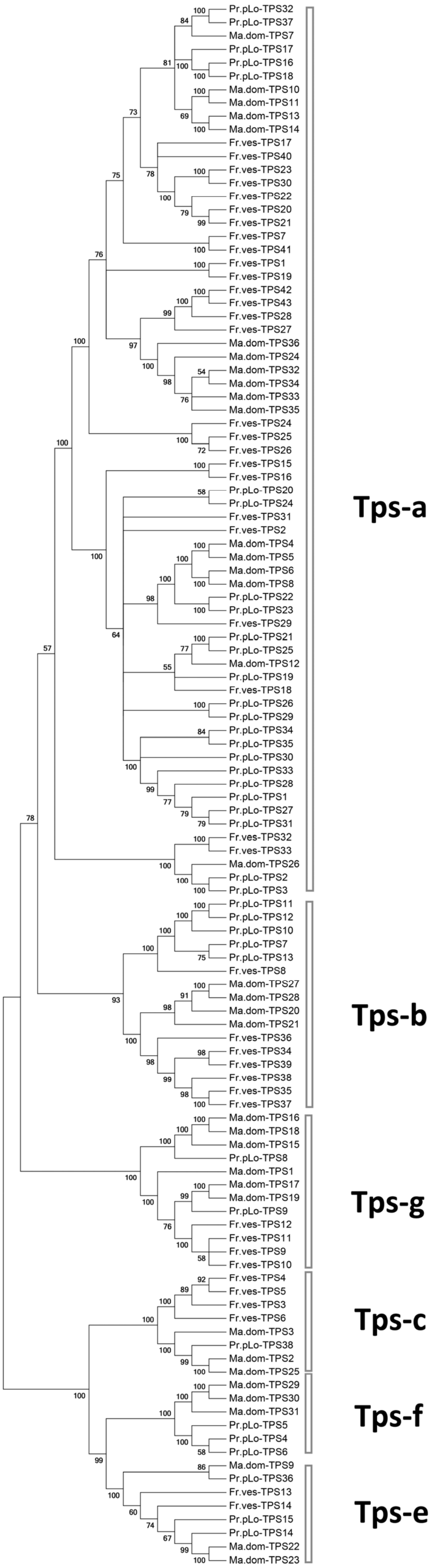

Figure S1. A Neighbor-joining phylogenetic tree of TPSs of the TPS proteins in three Rosaceae species (*P. persica, M. domestica, F. vesca*).

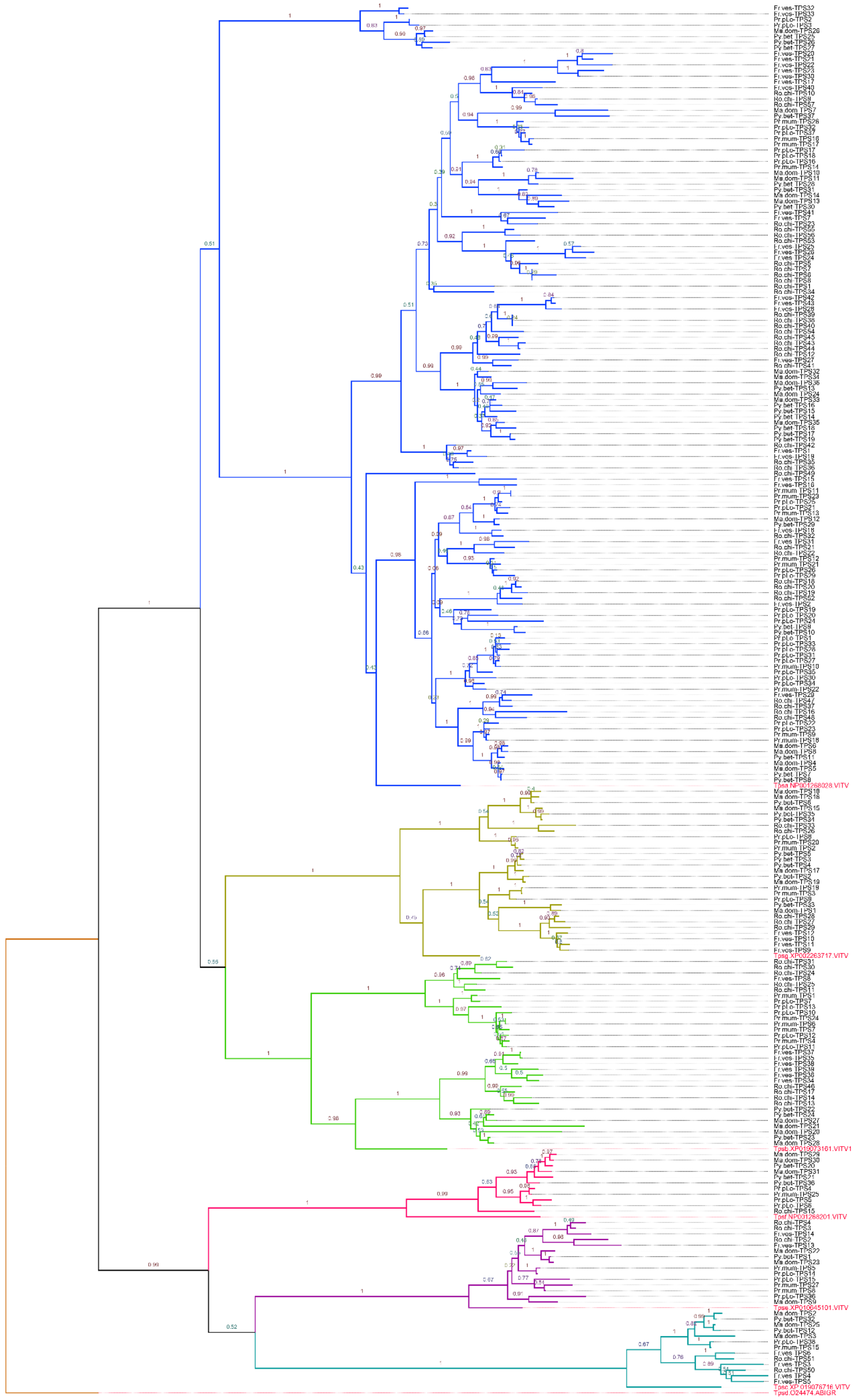

Figure S2. A maximum-likelihood phylogenetic tree of six representative Rosaceae species TPS gene family. The branches of TPS-a, TPS-g, TPS-b, TPS-c, TPS-e and TPS-f clades are colored in blue, yellow-green, green, red, purple and cyan, respectively.

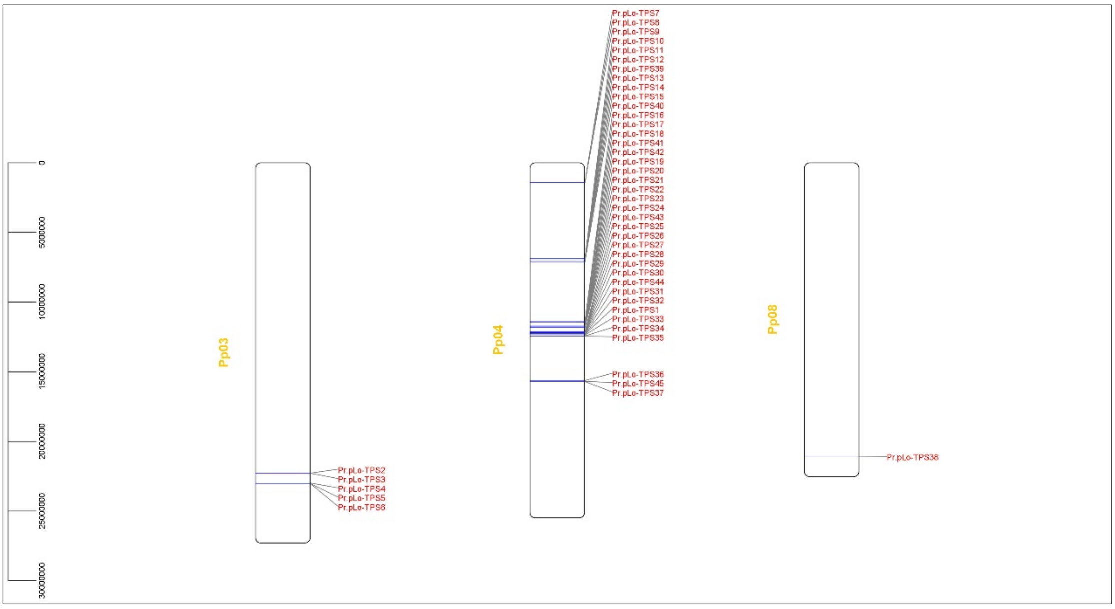

Figure S3. Chromosomal locations of TPS family members in peach 'Lovell' genome. The gene location visualize package of TBTools was used to exhibit chromosomal locations of the TPS genes. The number to the left of each chromosome represented the size of the chromosome in bp.

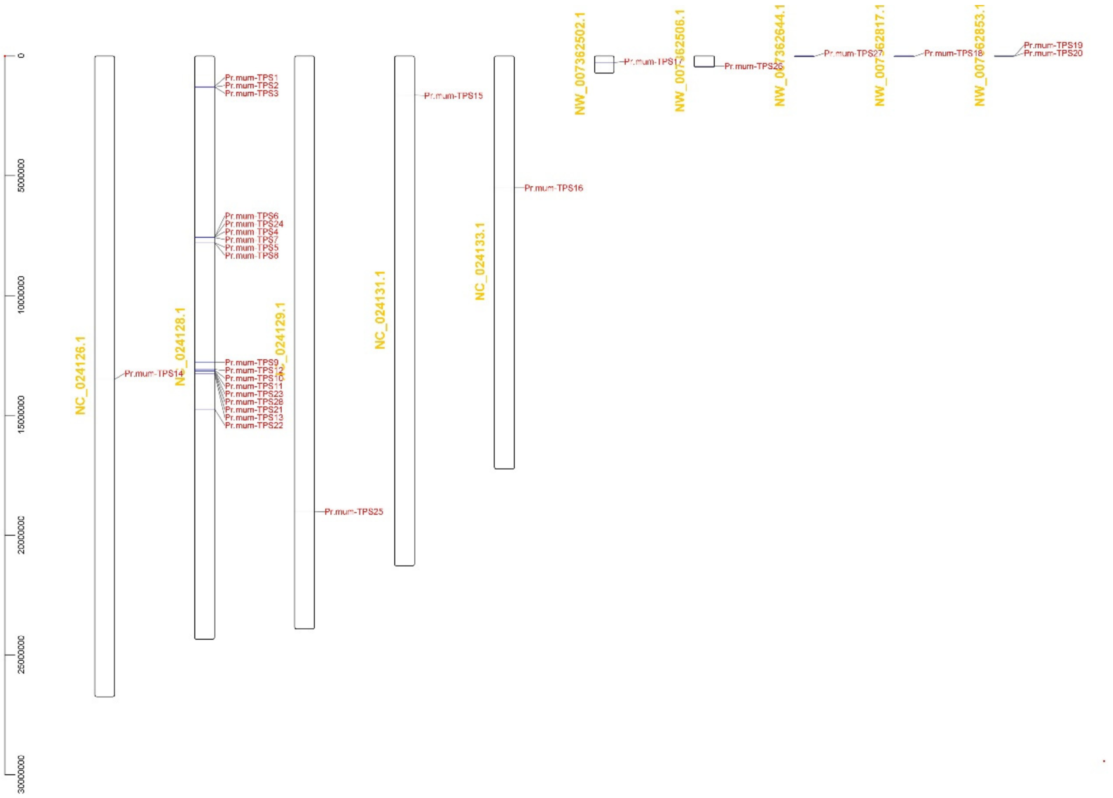

Figure S4. Chromosomal locations of TPS family members in *Prunus mume* genome. The gene location

visualize package of TBTools was used to exhibit chromosomal locations of the TPS genes. The number to the left of each chromosome represented the size of the chromosome in bp.

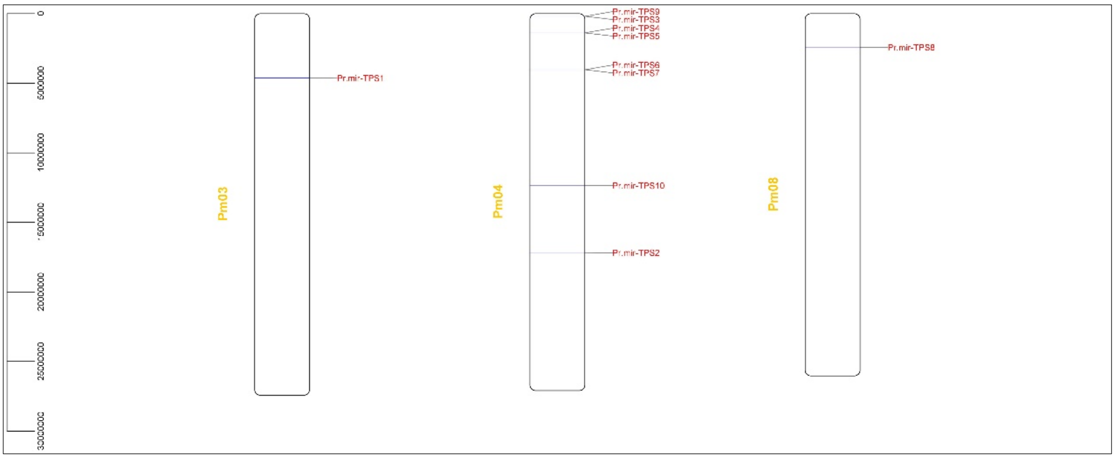

Figure S5. Chromosomal locations of TPS family members in *Prunus mira* genome. The gene location visualize package of TBTools was used to exhibit chromosomal locations of the TPS genes. The number to the left of each chromosome represented the size of the chromosome in bp.

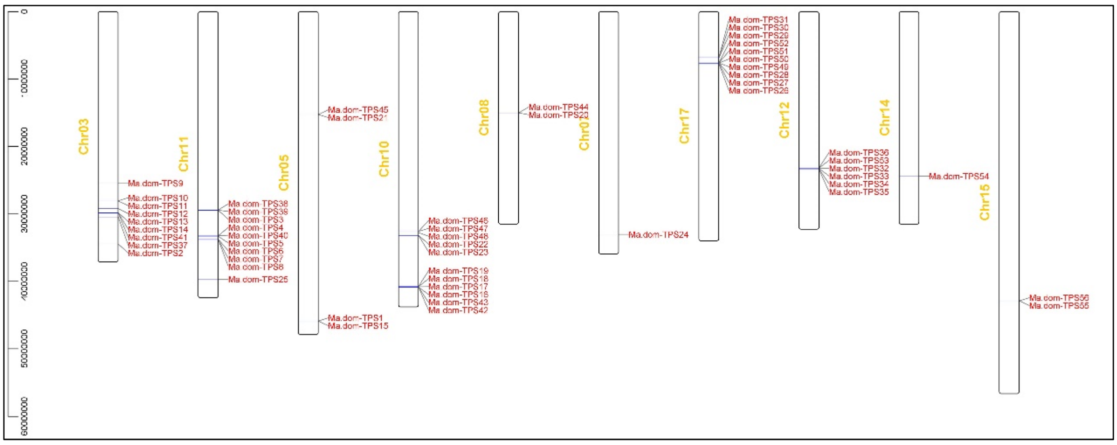

Figure S6. Chromosomal locations of TPS family members in *Malus x domestica* genome. The gene location visualize package of TBTools was used to exhibit chromosomal locations of the TPS genes. The number to the left of each chromosome represented the size of the chromosome in bp.

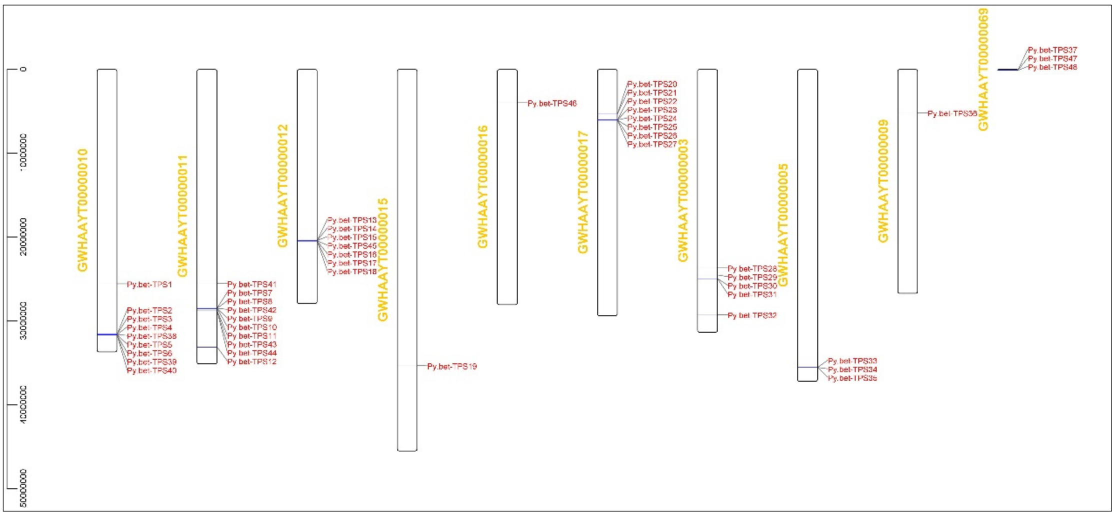

Figure S7. Chromosomal locations of TPS family members in *Pyrus betulifolia* genome. The gene location visualize package of TBTools was used to exhibit chromosomal locations of the TPS genes. The number to the left of each chromosome represented the size of the chromosome in bp.

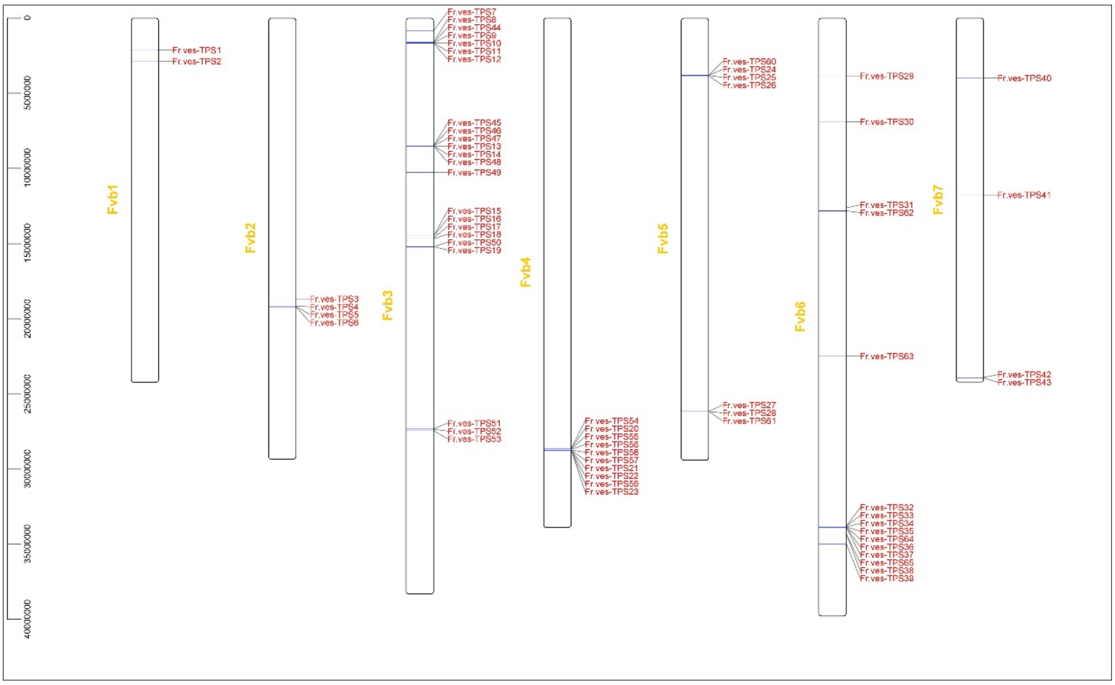

Figure S8. Chromosomal locations of TPS family members in *Fragaria vesca* genome. The gene location visualize package of TBTools was used to exhibit chromosomal locations of the TPS genes. The number to the left of each chromosome represented the size of the chromosome in bp.

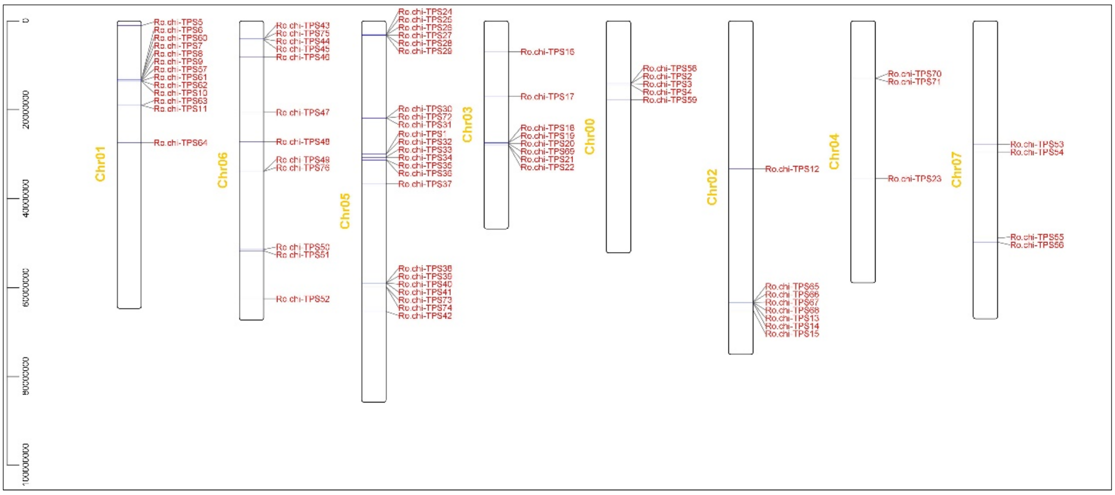

Figure S9. Chromosomal locations of TPS family members in *Rosa chinensis* genome. The gene location visualize package of TBTools was used to exhibit chromosomal locations of the TPS genes. The number to the left of each chromosome represented the size of the chromosome in bp.

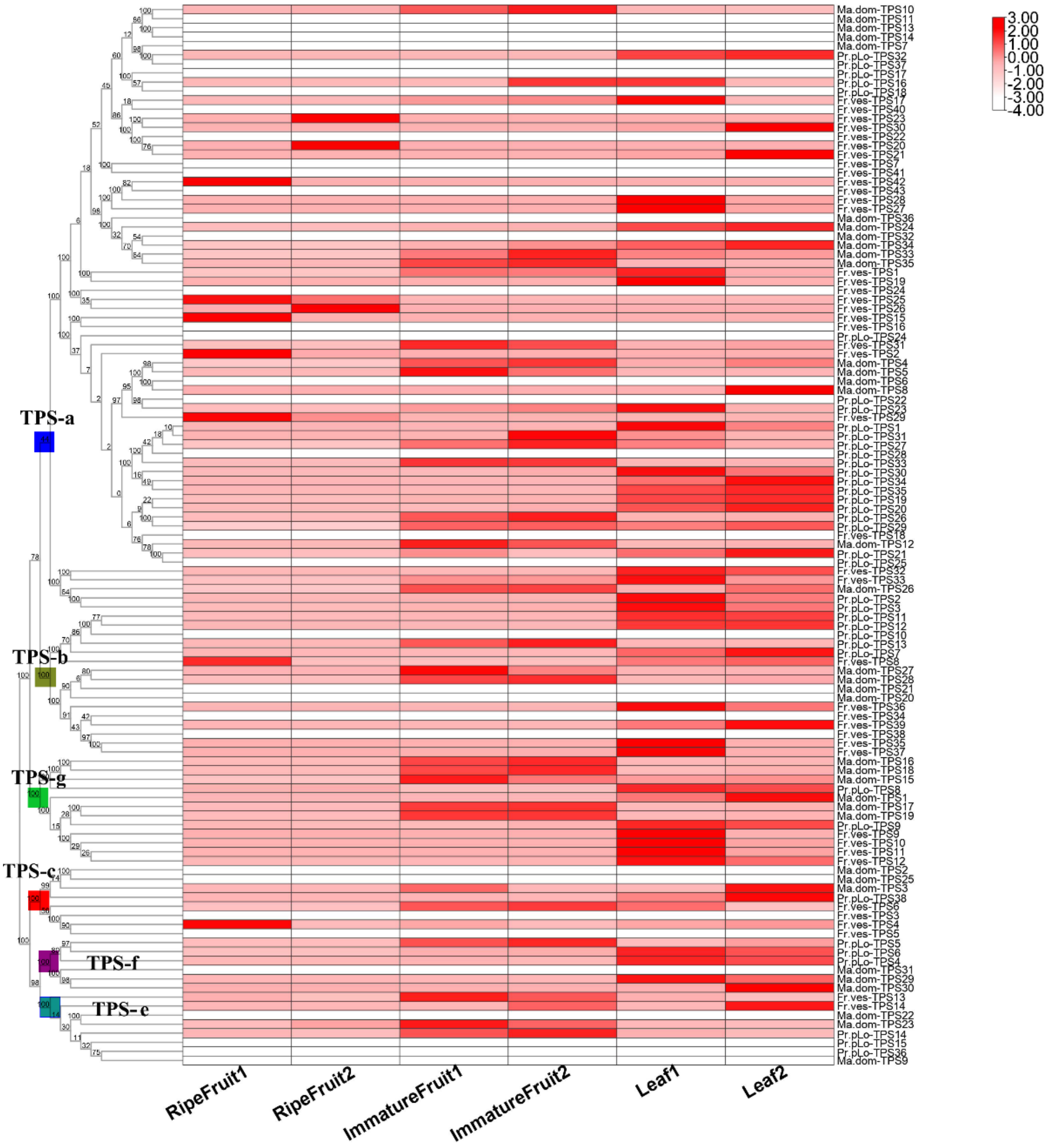

Figure S10. Expression pattern of TPS genes in three Rosaceae plants (*P. persica, M. domestica, F. vesca*). The x-axis represents different samples (ripe fruit, immature fruit and leaf), the y-axis represents TPS genes. There are two replicates for each tissue. The phylogenetic tree is shown on the left panel, the root nodes of TPS-a, TPS-g, TPS-b, TPS-c and TPS-e, TPS-f clades are indicated by blue, green, yellowgreen, red, benzo, and purple, respectively.
